# Low-cost high performance piezoelectric fabrics based on Nylon-6 nanofibers

**DOI:** 10.3389/fchem.2024.1525034

**Published:** 2024-12-04

**Authors:** Dong-Jun Kwon, JoAnna Milam-Guerreroa, Yun Young Choi, Nosang Vincent Myung

**Affiliations:** ^1^ Department of Chemical and Biomolecular Engineering, University of Notre Dame, Notre Dame, IN, United States; ^2^ Department of Materials Engineering and Convergence Technology, Research Institute for Green Energy Convergence Technology, Gyeongsang National University, Jinju, Republic of Korea

**Keywords:** piezoelectric, nanogenerator, electrospinning, nanofiber, e-textile, smart fabric

## Abstract

To fully harness the potential of smart textiles, it is cruical to develop energy harvesters which can function both as fabric and energy generator. In this work, we present a high performance low-cost piezoelectric nano-fabric using even-number Nylon (*i.e.,* Nylon-6). Nylon-6 was chosen for optimal mechanical properties such as mechanical strength and stiffness. To maximize the voltage output, Nylon six nanofibers with varying diameter and crystallinity were synthesized by adjusting the polymer precursor and solvent, along with electrospinning parameters, followed by post thermal treatment. The average diameter of electrospun nanofibers was finely tuned (down to 36 nm) by adjusting solution polymer precursor content and electrospinning parameters. The content of desired piezoelectric-active γ crystal phase enhanced upto 76.4% by controlling solvent types and post thermal annealing. The highest peak to peak voltage (V_33_) of 1.96 V were achieved from γ-phase dominant (>60%) Nylon-6 nanofiber fabric which has an average nanofiber diameter of 36 nm with high fiber fraction (*i.e.,* > 98%). Unlike its thin film counterpart, piezoelectric electrospun nanofiber fabric demonstrated durability against wear and washing. This work paves a new way to utilize Nylon-6 nanofibers in next-generation electronic textiles.

## 1 Introduction

Smart clothes, also known as smart wear, electronic textiles, or smart fabrics, are a next-generation apparel with extra functionality (Bhuvaneshwari, 2021; Cho et al., 2022). By harnessing the latest developments of advanced bio (chemical) sensing and flexible electronics, smart clothes can revolutionize personal healthcare by providing real-time sensing of physiological parameters including vital signs, body temperature, motion, and biomarkers (Hu et al., 2022; Yang et al., 2022; Lianori et al., 2022). With multifunctional, flexible devices becoming more ubiquitous, it is necessary to improve the energy efficiency of these materials thereby optimizing their energy utilization and storage properties (Magno et al., 2020; Longa et al., 2022). To improve these energy storage capabilities, the integration of energy generators into smart clothes is essential. That is to say, smart clothes require an energy harvesting function that is lightweight and can generate energy throughout the day potentially using human activities such as mechanical movement (Li et al., 2022a; Khan and Khan, 2022; Zou et al., 2022).

One of the most suitable energy-generating sources for smart clothes is the conversion of mechanical energy, generated by human movement, into electrical energy (Qi et al., 2022; Fang et al., 2022). A representative concept is the piezoelectric effect, in which mechanical stress generates an electric charge and *vice versa*, or the triboelectric effect, which generates electricity due to mechanical friction on the surface of the material (Zhu et al., 2013; Seol et al., 2014; Li D. et al., 2022; Sharma et al., 2022). Mechanical stresses, such as cyclic compressive, tensile, or frictional stresses could potentially generate sufficient energy to power small handheld devices from everyday human movement (Hatcher, 2023; Jiang et al., 2022; Lv et al., 2022). Regrettably, the sensitive fabric is prone to contamination during war, and the current low durability of smart clothes results in poor washing fastness.

To address the wash and mechanical durability limitations of energy generators for smart clothes, it is more practical to use a polymer currently employed in textile for clothing as energy generator (Zhang et al., 2021; Ghosh et al., 2017; Shawon et al., 2021). Commonly used textiles, such as polyurethane, nylon, polyester, and cotton, meet the minimum physical properties for use as clothing (Zeong et al., 2014; Crane and Bovone, 2006); however, to be used as an energy generator for smart clothes, the fiber shape must be easily applied to clothing where it is thin and lightweight. Fabrics made out of nanofibers might be the optimum morphology providing a thin, lightweight sample while potentially maximizing piezoelectric responses. Nylon is one of the most cost-effective textile materials (Burkinshaw and Maseka, 1996) with its superior mechanical strength, excellent washing fastness (Wu et al., 2017), easy nanofiber processing (Dhineshbabu et al., 2014), and effective polarization performance due to its piezoelectricity due to the influenced by amide bonds (Newman et al., 1977).

Within the Nylon class, there are several structural (*e.g.,* even-numbered Nylon and odd-numbered Nylon) and conformational phases (*e.g.,* α- and γ-phase) which result in different piezoelectric properties. While the α-phase is mechanically strong due to hydrogen bonding caused by amide bonds in an alternating anti-parallel planar configuration, it is piezoelectrically inactive as those same alternating amide bonds cancel out any polarization (Stephens et al., 2004). Traditionally processed even-numbered Nylons, (*e.g.,* Nylon-6, Nylon-66), have a high α-phase content and therefore they exhibit limited piezoelectric properties (Mit-uppatham et al., 2004). Furthermore, the α-phase strongly induces hydrogen bonds, forming intermolecular packing and thus limiting the dipole movement of molecules, which disturbs or prevents polarization (Newman et al., 1980). For traditionally processed odd-numbered Nylons, (*e.g.,* Nylon-7, Nylon-11), the γ-phase is known as the dominant phase that can be induced to have a greater piezoelectric response (Scheinbeim, 1981; Newman et al., 1990). In these structures, the free volume of the γ-phase is higher than that of the α-phase, and the molecular chain movement generated by external stimuli increases the piezoelectric effect (Takase et al., 1991).

This work explores the hypothesis that the crystal structure of Nylon-6, which is most widely used for general clothing, can be controlled through solvent choice during electrospinning subsequently made into an energy generator for smart clothes. By inducing the desired piezoelectric-active γ crystal phase through detailed experimental designs, we demonstrate a low-cost piezoelectric nanogenerator material capable of withstanding wear and washing. Finally, for Nylon six nanofibers, the piezoelectric properties remained stable even after washing, with crystal phase changes showing greater stability compared to Nylon six film. This study confirms that optimized Nylon six nanofibers provide an ideal composition for energy generation in smart wear applications.

## 2 Materials and methods

### 2.1 Materials

Nylon six pellets were purchased from Sigma-Aldrich. Formic acid 85% and 97% (F85 and F97, respectively), Trifluoroacetic acid (TFA) and 1,1,1,3,3,3-Hexafluoro-2-propanol (HFIP) were purchased from Fisher Scientific. The BYK-377 (hereafter referred to as BYK) surfactant was obtained from BYK Additives and Instrumentation. All materials were used without further treatment.

### 2.2 Solution preparation

Electrospinning solutions were prepared by weighing 4.8–20 wt% of Nylon pellets and were dissolved in each of the following four solvents (*i.e.,* formic acid 85%, formic acid 97%, acetone and trifluoroacetic acid (TFA) 60:40 mol%, and HFIP:TFA 75:25 mol%). The Nylon pellets were soaked and stirred until homogeneous at room temperature. The Nylon film was prepared by drop-casting with an average film thickness of 90–100 μm.

### 2.3 Solution characterization

Viscosity measurements were performed using a CPA-40 spindle connected to a Brookfield DV2THB viscometer. The solution viscosity was determined to be independent of the shear rate. The viscosity values were measured at a 95% torque at each rotational speed starting at 0.5 rpm–200 rpm. An automatic surface tensiometer (Shanghai Fangrui Instrument, QBZY-1) with platinum-coated plate was used to measure the surface tension. Solution electrical conductivity was measured using an electrical conductivity probe from pH Bench multi-parameter (Ohaus, Starter 3100M). All solution property measurements were taken at room temperature immediately before electrospinning to more closely correlate them to the resulting nanofiber properties.

### 2.4 Electrospinning

The prepared Nylon solutions were drawn into a 5-mL BD Luer Lok syringe with a blunt needle with an inner diameter of 0.24 mm and loaded onto a syringe pump (New Era, NE100). The needle tip was set at a 15 cm distance from the drum collector. The electrospinning conditions, such as voltage and feed rate, were altered depending upon the solution properties to produce the optimal Nylon-6 nanofibers with sufficiently high fiber fraction and low average fiber diameter. The voltage was regulated between 7.7 and 29.0 kV and the feed rate was regulated between 0.1 and 5 mL/h. The grounded drum collector was wrapped tightly with aluminum foil and rotated at 300 rpm. Environmental conditions were kept at 23°C ± 2 C and absolute humidity of 0.008 ± 1 kg water/kg dry air (∼40% relative humidity at 23 C).

### 2.5 Nanofiber characterization: Physical properties and piezoelectric properties

Morphology of the as-spun nanofiber and the composite materials were observed with a scanning electron microscope (Prisma E SEM, ThermoFisher Scientific, USA). Prior to analysis, a thin layer of gold was sputtered using Electron Microscopy Sciences 575X over the samples at 20 mA for 30 s to minimize surface charging. Obtained SEM images were imported to ImageJ software to measure the average fiber diameter, which was obtained by measuring the diameter of 30 unique nanofibers. The bead density was calculated by dividing the total number of beads from a single SEM image by the total area of the image. Fiber fraction was determined by the proportion of nanofibers in the total product, which could include beads and clumps. The molecular and crystal structures were observed throughout experiments with a Perkin Elmer Spotlight 200 Fourier-transform infrared (FT-IR) spectrometer and a Bruker D8 Discover X-ray diffractometer (XRD), respectively. For FT-IR, approximately 0.5 × 0.5 cm2 of nanofiber mat was cut and placed in a pass-through sample holder. FT-IR spectra were then obtained by scanning from 400 to 4000 cm-1 at a resolution and scan increment of one and 0.5 cm-1, respectively. For XRD, multiple 2 × 2 cm2 pieces were coherently layered to ensure X-ray detection of the sample. Data collection was performed at room temperature at *λ* = 1.5406 Å over a 2θ range of 15°–30° with a step size of 0.025°. Polarization was confirmed to confirm the ferroelectric properties of Nylon nanofibers according to the respective solvents. A Radiant multiferroic meter (RT66C) was used by applying 1, 5, 10, 20, and 50 V to measure the resulting ferroelectric hysteresis loop.

Cantilever measurements were performed on nanofiber samples prepared as described by Supplementary Figure S1 and previously reported in literature (Ico et al., 2016; Tai et al., 2021). Two electrodes were made for each nanofiber sample to measure the voltage vertical to the direction of fiber length (denoted as V_33_). A 7.2 × 1.6 × 0.01 cm^3^ brass shim was first covered on both sides with polyimide tape for electrical isolation, then one side of the shim was exposed over 4 × 1.2 cm^2^ at the center of the brass shim as a contact pad for the nanofiber sample. Nanofiber samples of 4 × 1.2 cm^2^ size were cut and then fixed to the opening with a piece of double-sided copper tape, which served as the bottom-contact electrode. The other side of the nanofiber was in contact with the brass shim without an opening and thus was electrically insulated. A pair of 24-gauge wires were soldered to the two brass electrodes, sealed with polyimide tape, then connected to a breadboard with inputs to a Picoscope 2204A™ (Pico Technology Ltd.) to measure the absolute output voltage from the nanofiber mats. V_33_ results were obtained through the cantilever test where the V33 value provides a benchmark of comparison for piezoelectric performances.

Changes in the piezoelectric properties of Nylon films and nanofibers were observed upon washing where Nylon six nanofibers and film were placed in a beaker containing 200 mL of water and placed in an ultrasonic bath (SPW Industrial, Branson 3,510 Ultrasonic Cleaner Water Bath). The ultrasonic bath ran for 1 h at 30°C and the samples were dried at 80 C for 2 h to remove all the moisture. Stability testing on the piezoelectric properties was performed in 15 days intervals over a 45 day period. In addition, the V_33_ of Nylon nanofiber was investigated according to the change in elongation of Nylon nanofibers. Based on these results, the applicability of Nylon nanofibers for smart wear was reviewed.

## 3 Results and discussion

### 3.1 Effect of solvents on the electroactive *γ*-phase in Nylon-6 fibers

**FIGURE 1:**
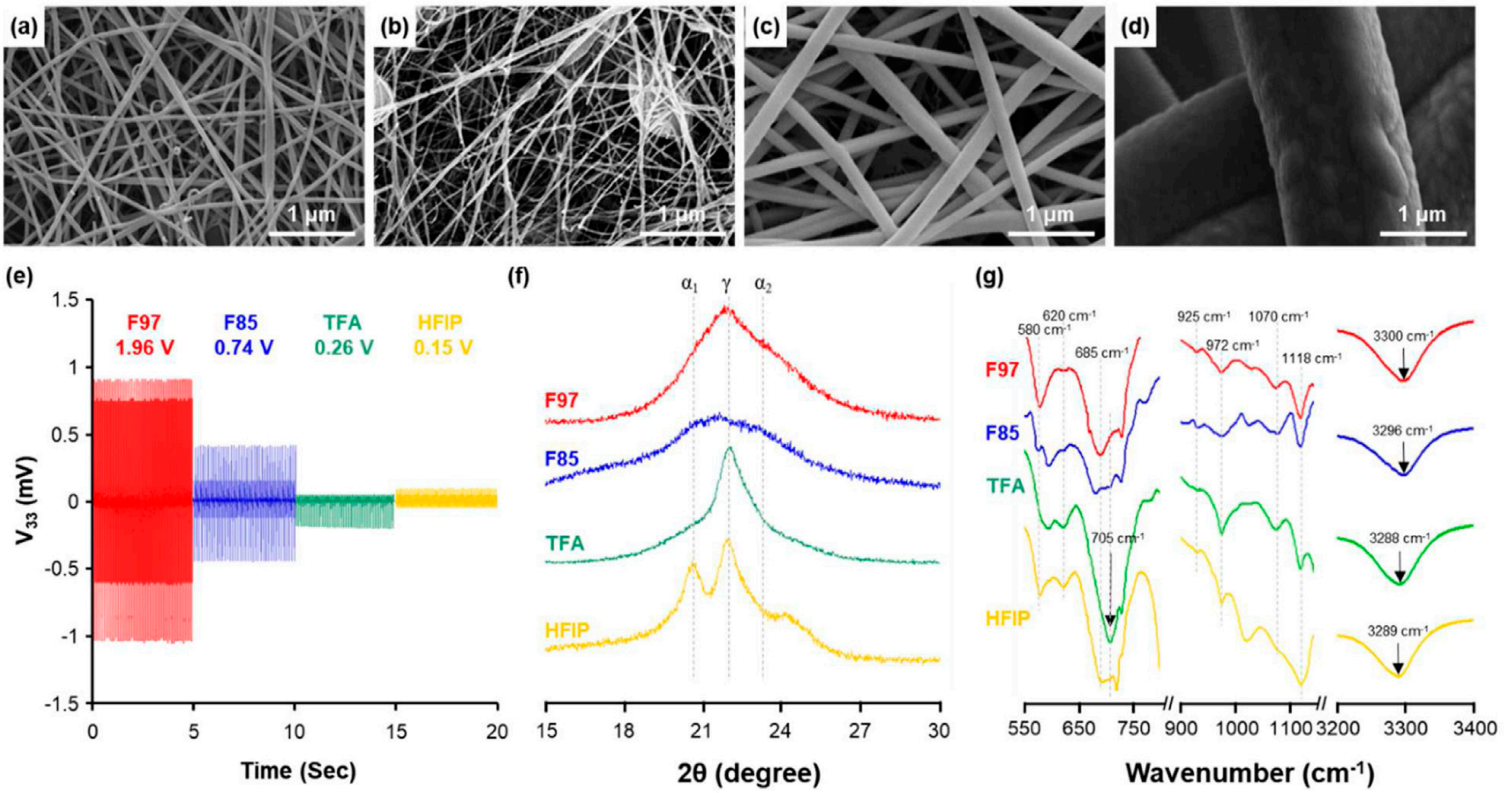
SEM image of Nylon-6 nanofibers with different solvents: **(A)** formic acid 97% (F97); **(B)** formic acid 85% (F85); **(C)** Trifluoroacetic acid:acetone (60:40 mol%, TFA); **(D)** 1,1,1,3,3,3-Hexafluoro-2-propanol:Trifluoroacetic acid (75:25 mol%, HF). The content of polymer was the same at 10 wt%. **(E)** Piezoelectric voltage output (V_33_) of as-spun Nylon-6 nanofibers with various solvents. **(F)** XRD patterns of as-spun Nylon-6 nanofibers with different solvents type from 2θ of 15 to 30^o^ with the allowed reflection peaks. **(G)** FT-IR spectra of Nylon-6 nanofibers.

To study the effects of solvent type upon the electrospun nanofibers, the concentration of Nylon-6 was fixed at 10 wt%. As seen in Table 1, the solution properties (*e.g.,* viscosity, surface tension and electrical conductivity) varied depending upon the solvent used although Nylon concentrations were fixed. The difference in solution properties of solvents had a significant effect upon the fiber diameter and fiber fraction of electrospun nanofibers as well. For formic acid, the resulting average fiber diameter was <40 nm with smooth surfaces, as shown in Figure 1A. The fiber fraction was different depending upon the purity of the formic acid. For example, a lower purity of formic acid (*i.e.,* 85%) resulted in a higher occurrence of beads and clump generations as well as a significantly lower fiber fraction of 50.1% (Figure 1B). In the case of TFA, the average fiber diameter increased to 172 nm with a homogeneous nanofiber sheet with a high fiber fraction >99%. When HFIP was used as a solvent, a relatively high viscosity of 2,178 cP was obtained that resulted in microfibers being produced during electrospinning. While the average fiber diameter increased, the fiber fraction was low due to fibers being intermittently melted from the influence of the solvent. Both TFA and HFIP based electrospun solutions resulted in nanofibers with a higher average fiber diameter of 172 nm to the micrometer range (Figures 1C, D).

**TABLE 1:** Solution properties of Nylon-6, and Diameter of Nylon six nanofiber with the different solvents.

Case	Viscosity (cP)	Surface tension (mN/m)	Conductivity (mS/cm)	Fiber diameter (nm)	Fiber fraction (%)
F97	145	38.8	12.3	36 (13)	97.9
F85	126	43.8	11.8	25 (17)	50.1
TFA	205	26.6	7.1	172 (56)	99.3
HF	2178	51.2	5.9	1,775 (267)	73.1

The change in the crystal structure of Nylon-6 nanofibers depending upon the solvent can be seen in the XRD results (Figure 1F). For Nylon-6, the piezoelectric inactive phase is referred to as the *α*-phase with two prominent X-ray peaks at 20° and 23° while the desired, piezoelectric-active *γ*-phase manifests as a single peak around 22°. Table 2 shows the relative percentages integrated for *α*- and *γ*-phases when varying the solvents. For Nylon, it has been established that an odd numbered carbon chain is required for piezoelectric properties; however, the increased *γ*-phase ratio for average fiber diameter 30 nm shows that even numbered Nylon-6 can also display piezoelectric properties.

**TABLE 2:** Peak Location and d-Spacing Based on XRD of the As-Spun Nylon-6 Nanofibers.

Type	Peak location (2θ, °)	*d*-spacing (nm)	Grain size (nm)	Area integrated % (%)
F97	γ: 21.98	0.404	5.59	61.8
	α: 20.82	0.426	6.59	13.0
	α: 24.18	0.368	4.49	25.2
F85	γ: 21.53	0.406	3.92	48.7
	α: 18.40	0.482	7.02	13.2
	α: 23.23	0.375	3.33	38.1
TFA	γ: 22.03	0.403	13.04	48.5
	α: 20.03	0.443	7.43	14.2
	α: 23.18	0.383	4.65	37.0
HF	γ: 21.98	0.404	17.04	43.1
	α: 20.65	0.430	15.61	32.7
	α: 23.97	0.371	11.63	24.2

To support the supposition that the Nylon-6 crystalline phase was solvent induced, similar solvent variation studies were performed upon drop cast films. Varying the solvent used to make the Nylon film via dropcast method resulted in changes in the crystal phase and piezoelectric properties, as seen in Supplementary Figure S2. Nylon-6 thin films are known to have both *α*- and *γ*-phases present, but the non-piezoelectric *α*-phase content was dominated compared to *γ*-phase for dropcast Nylon-6 films. Supplementary Figure S2C shows that the Nylon-6 thin film made with formic acid 97%, which showed the highest piezoelectric properties, had approx. 34% of *γ*-phase while other solvents showed an average of 15% *γ*-phase. While electrospun Nylon-6 fiber with TFA and HFIP have a strong *γ* peak present, the relative *α* peaks present overshadow the integrated area of *γ* peaks, as shown in Table 2. Thus, despite the synthetic technique used, it was concluded that the crystal phase of Nylon-6 could be controlled by varying the solvent. Consequently, the piezoelectric active phase of Nylon-6 is concurrently induced by both solvent effects and the applied voltage when electrospinning.

Supplementary Figure S3 shows that there is no discernable difference in the ferroelectric polarization hysteresis of Nylon nanofibers by varying the solvent; however, Supplementary Figure S4 confirms that the piezoelectric properties of Nylon-6 are proportional to the *γ*-phase ratio and inversely proportional to the average fiber diameter.

FT-IR data further supports the chemical structures determined by XRD. Supplementary Table S2 summarizes potential peaks that can be assigned to the *α*- and *γ*-phases for Nylon-6 (Tu et al., 2020; Nail et al., 2006). For all electrospun Nylon-6 nanofibers, both *α* and *γ* peaks of different intensities were present regardless of which solvent was used. It was confirmed that using formic acid 97% as a solvent formed a strong hydrogen bond compared to other solvents through the N-H hydrogen bonding peak observed at 3,300 cm^-1^ (Rwei et al., 2019; Eom et al., 2021). It might be attributed to that the strong intermolecular structure by hydrogen bonding activates the polarization action by external stress, thereby enhancing piezoelectric properties of Nylon-6 nanofibers (Scheinbeim, 1981; Choi et al., 2020). Based on the XRD and FT-IR results, Nylon-6 nanofibers that exhibit higher *γ* peak ratio and strong hydrogen bonding display an applicable piezoelectric effect.

The voltage output of these electrospun nanofibers were tested using a cantilever apparatus vibrated at 10 Hz at various decibels. Previous studies have shown that the average fiber diameter and the piezoelectric effect display an inverse relationship (Yu et al., 2022). The electrospun Nylon-6 nanofibers with formic acid 97% (F97) resulted in the smallest average fiber diameter with the fiber fraction over 95%, which we defined as a threshold for nanofibers with minimum defects. As seen in Figure 1E, Nylon-6 nanofibers with F97 produced the highest peak to peak voltage output of 1.96 V.

### 3.2 Optimization of Nylon-6 nanofibers

From this point forward all solutions were made with formic acid 97% to further study the effects of other electrospinning conditions (Table 3). When the concentration of Nylon-6 increased from 8 wt% to 10 wt%, a significant increase in the viscosity occurred from 62 cP to 145 cP, respectively. To further understand how the electrospinning conditions affect the resulting nanofiber properties, the electrospinning voltage was also varied between 15 kV and 25 kV. The solution feed rate varied between 0.3 mL/h and 0.5 mL/h.

**TABLE 3:** Solution and nanofiber properties of F97. All electrospinning and environmental conditions were fixed.

Case	Viscosity (cP)	Applied Voltage (kV)	Feed rate (mL/h)	Fiber Diameter (nm)	Bead Fraction (%)	Fiber Fraction (%)
1	62	15	0.3	53 (16)*	13	87
2		15	0.5	62 (17)	6	94
3		25	0.3	43 (15)	23	77
4		25	0.5	48 (15)	19	81
5	145	15	0.3	40 (7)	1	99
6		15	0.5	50 (12)	3	97
7		25	0.3	36 (13)	2	98
8		25	0.5	44 (6)	4	95

*Standard deviation.

When the solution was injected too quickly, it resulted in sections of melted nanofibers, as seen in Supplementary Figure S5C. When the applied voltage exceeds a certain threshold, the charged jets are ejected from the Taylor cone that results in the electrospinning technique. Taylor cones have a significant effect upon electrospun nanofibers as the charged jets from the Taylor cones produce bead-free nanofibers, as seen in Supplementary Figure S6. When the applied voltage was lower than the threshold, solution droplets resulted in electrospraying instead of electrospinning. Conversely, when the applied voltage was too high (>26 kV), fiber breakage was seen. For electrospinning of Nylon-6 with F97, the applied voltage was retained between 14.4 kV and 25 kV to create a stable Taylor cone.

**FIGURE 2:**
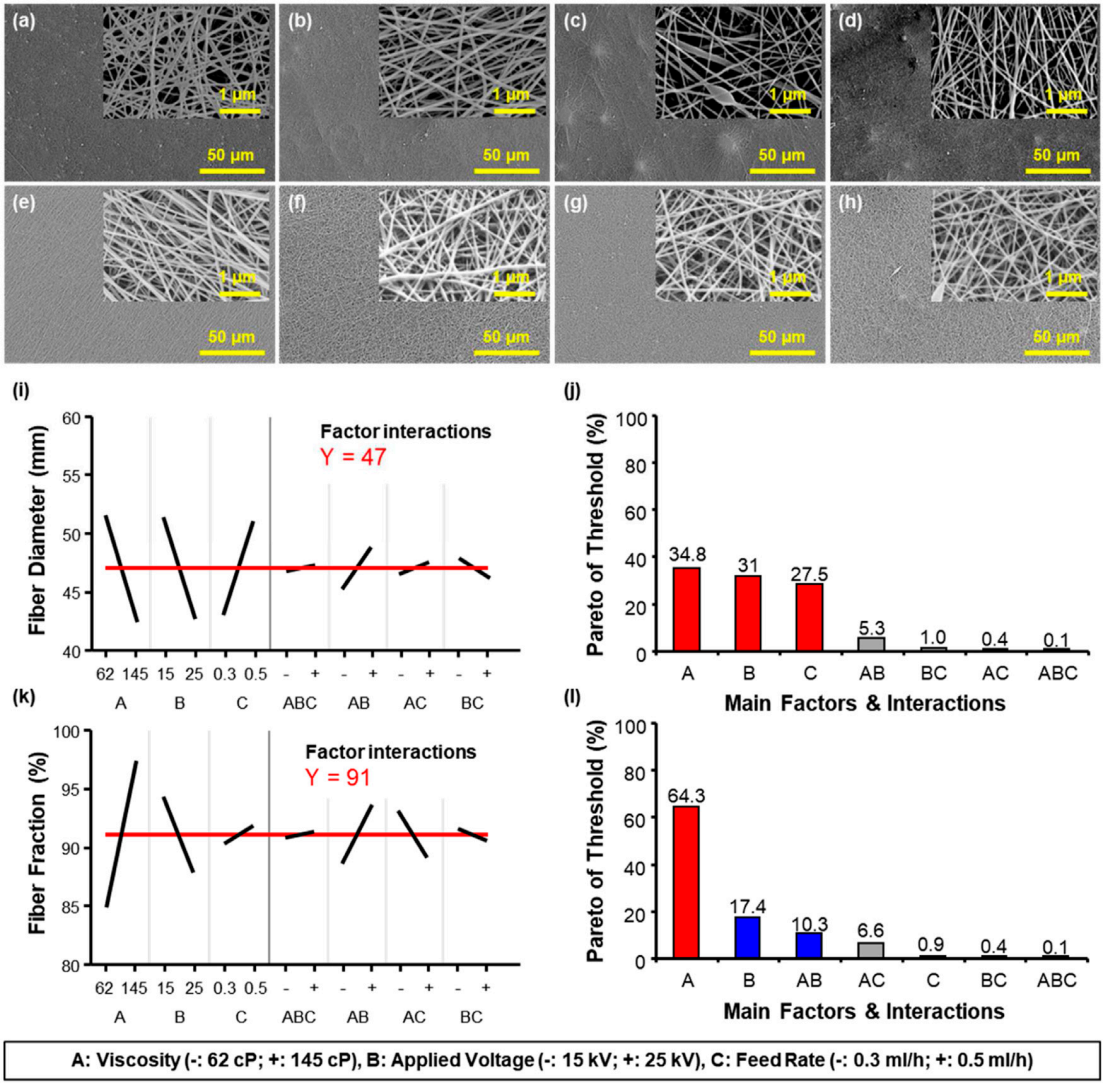
Effects of electrospinning solution properties on the morphology of Nylon-6 nanofibers. Respectively, of the electrospinning solution properties (see Table 2) SEM images of **(A)** 1, **(B)** 2, **(C)** 3, **(D)** 4, **(E)** 5, **(F)** 6, **(G)** 7, and **(H)** 8 cases for Nylon-6 nanofibers electrospun. Design-of-experiment (DOE) factor analysis and Pareto of threshold on **(I, J)** fiber diameter and **(K, L)** fiber fraction.

The experimental results listed in Table 3 are summarized in Figure 2 where they are shown as three factors of DOE analysis. Figures 2A–H shows SEM images of the effects of varying the applied voltage (kV) and solution spinning feed rate (mL/hr) where the average nanofiber diameters varied from 36 nm to 62 nm. The DOE analysis is shown in Figures 2I–L where the factors that were considered were A, B, and C that are viscosity of the solution, applied voltage, and feed rate, respectively. It was confirmed that the only variable significantly influencing the fiber fraction was viscosity. As confirmed in Figure 1E, when the bead generation rate of nanofibers is high, the voltage outputs are reduced regardless of how small the diameter of the fibers is. Thus, piezoelectric performance can be hindered by a high bead fraction present in the nanofiber mat.

**FIGURE 3:**
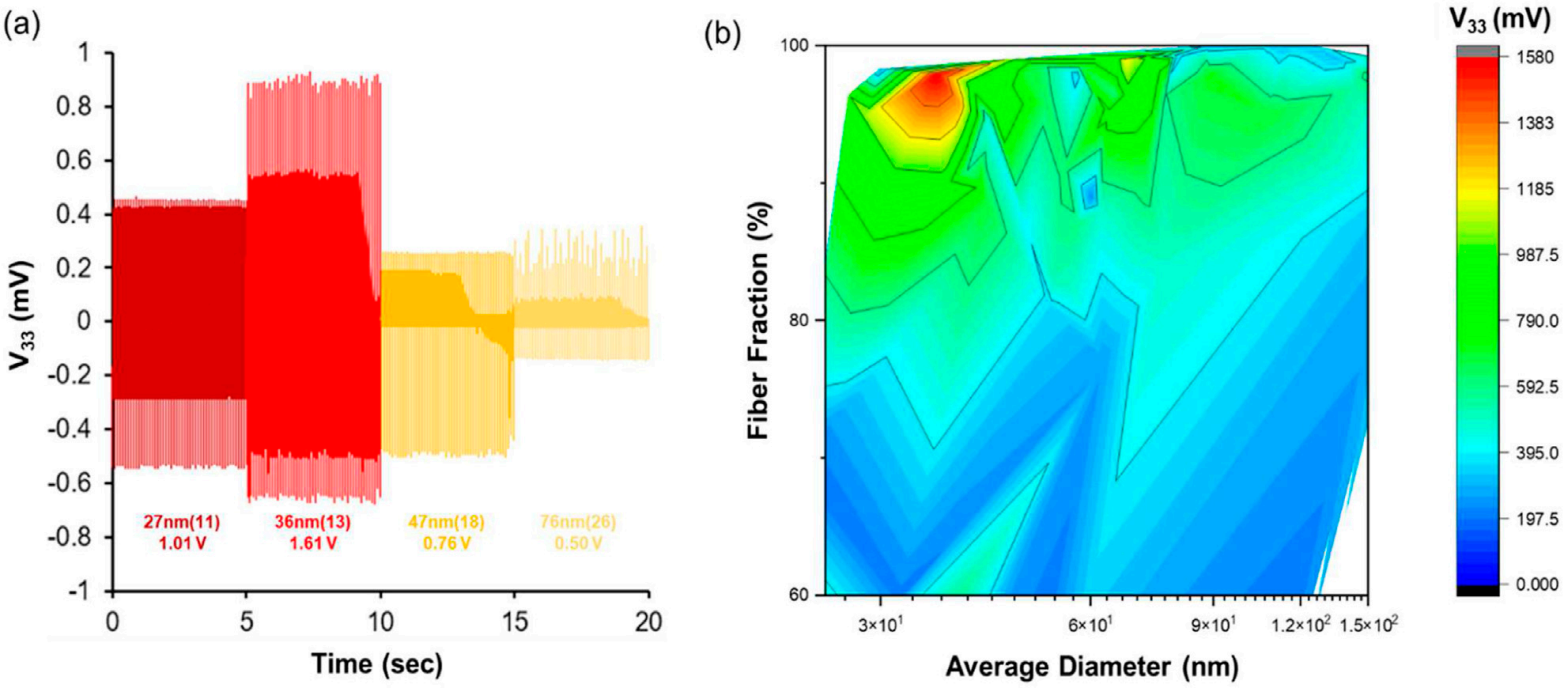
**(A)** Piezoelectric voltage output (V_33_) of Nylon-6 nanofibers with various diameters. **(B)** Changes in V_33_ according to fiber fraction and average diameter of Nylon-6 nanofibers.

Additionally, the bead fraction of the nanofiber mat could be reduced by controlling the surface tension of the solution. As shown in Supplementary Table S4, the values of Nylon-6 and BYK-377 (surfactant) concentration were confirmed to reduce bead generation by DOE analysis. As shown in Supplementary Figure S7, it was possible to control the beading according to the presence or absence of a surfactant such as BYK. To analyze the optimal composition of Nylon-6 nanofibers, we obtained Nylon-6 nanofibers with various process parameters (applied voltage, feed rate, collector rpm, solution property, etc.). The results of nanofiber diameter, fiber fraction, and V_33_ according to those parameters are summarized in a 3-D plot in Supplementary Figure S7 and a heat map in Figure 3B. Representative results are summarized in Figure 3. Through Figure 3A, it could not be explained conclusively that the lower the nanofiber diameter, the higher the V_33_ result. The reason being that when 27 nm class nanofibers were produced, the fiber fraction was as low as 80–90%, so the optimal piezoelectric properties could not be exhibited. However, when Nylon-6 nanofibers had an average diameter of 36 nm and the fiber fraction was greater than 90%, the highest V_33_ result was shown. Based on the results of Figure 3B, it was expected that the maximum V_33_ occurred under the conditions of the nanofibers having a diameter of 30–40 nm and high fiber fraction. In other words, it was found that nanofibers with optimal piezoelectric performance were synthesized only when the fiber fraction was increased while simultaneously reducing the fiber diameter.

### 3.3 Post thermal treatment to further enhance piezoelectric properties

**FIGURE 4:**
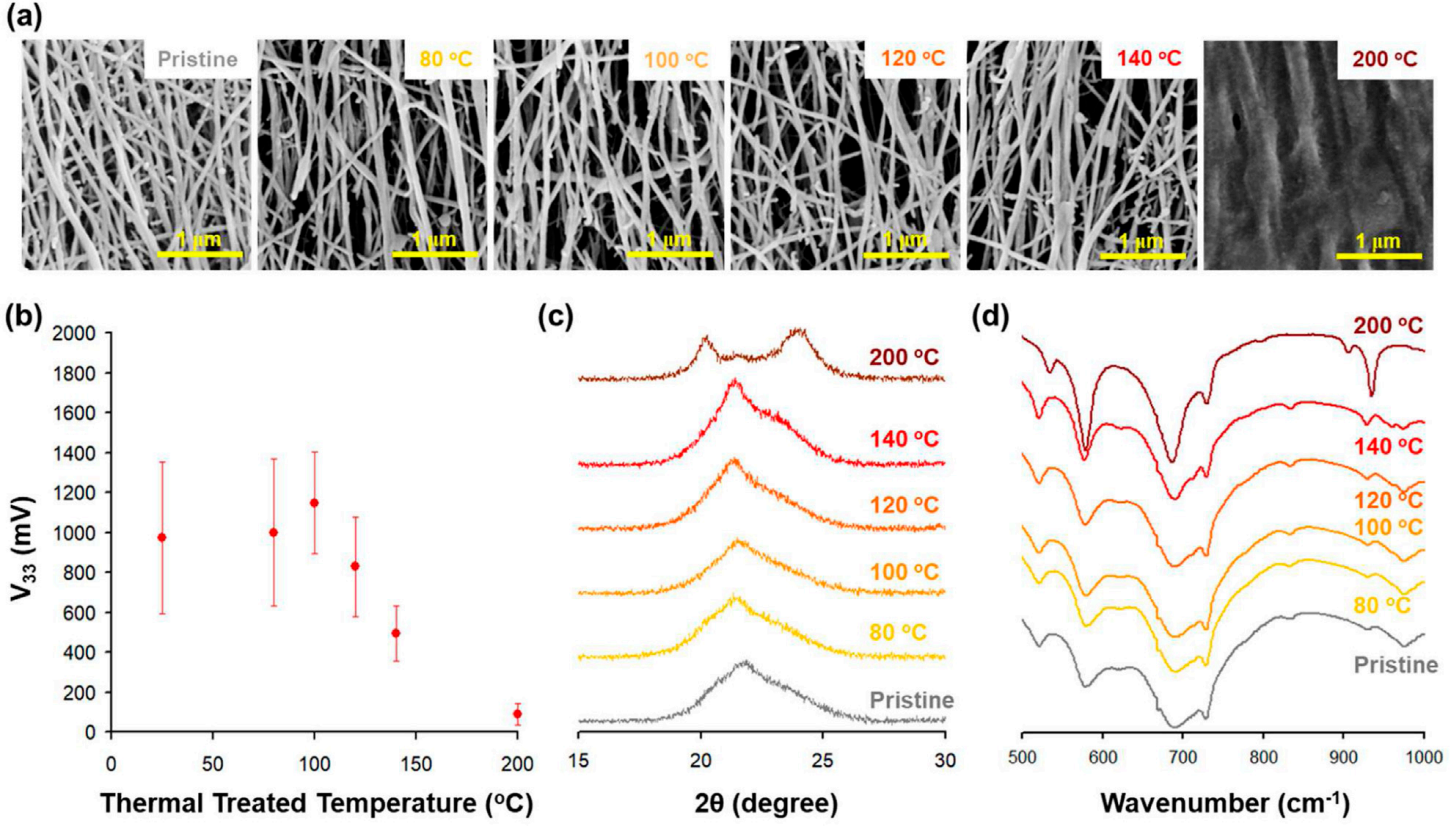
**(A)** SEM image of changes in nanofibers according to thermal treated temperature. **(B)** V_33_ of F97 with various thermal treated temperature. **(C)** XRD and **(D)** FT-IR of F97 with thermal treatment temperature.

Figure 4 summarizes the effects of different temperatures of post-thermal treatment on the electrospun nanofibers. The post-thermal treatment was carried out at 80, 100, 120, 140, and 200°C. Figure 4A shows that the shape of the nanofiber remains similar up to 140°C, but at 200°C deformation of the nanofibers can be observed. When cantilever testing was performed to obtain piezoelectric properties, the highest peak to peak voltage was exhibited when nanofibers were thermally treated at 100°C for 2 h (Figure 4B). The XRD data, in Figure 4C, shows that as the pristine nanofibers were thermally treated up to 100°C, the *γ* peak present at 22^o^ increased in intensity. As the thermal treatment temperature increased beyond 100°C, the *γ* peak starts to shift to lower 2θ indicating a larger unit cell such as that is seen with the *α* structure. At 120°C and at 140°C, the peak shift is significant and at 200°C two distinct *α* peaks can be seen. The *γ*-phase peak ratio was calculated by taking the area integrated (%) via peak deconvolution using OriginPro and the results are summarized in Table 4. These results show that from pristine to 100°C, the *γ*-phase peak increased from 59.1% to 76.4%. Beyond 100°C, the calculated *γ*-phase decreased significantly, and the *α*-phase increased with the increasing temperature.

**TABLE 4:** Peak Location and *d*-Spacing Based on XRD of the Nylon-6 nanofibers depending on the thermal treatment.

Type	Peak location (2θ, °)	*d*-spacing (nm)	Grain size (nm)	Area integrated % (%)
Pristine	γ: 21.69	0.409	8.01	59.1
	α: 20.53	0.432	13.14	8.5
	α: 23.49	0.378	7.61	32.4
80 ^o^C	γ: 21.33	0.416	6.90	73.9
	α: 20.41	0.435	13.80	4.2
	α: 23.48	0.379	8.17	21.9
100 ^o^C	γ: 21.41	0.415	6.76	76.4
	α: 20.45	0.434	12.34	3.2
	α: 23.33	0.381	8.20	20.4
120 ^o^C	γ: 21.33	0.416	11.29	36.8
	α: 20.31	0.437	9.76	14.1
	α: 22.94	0.387	5.59	49.1
140 ^o^C	γ: 21.48	0.416	16.07	23.6
	α: 20.35	0.436	9.35	27.0
	α: 22.93	0.387	6.77	49.4
200 ^o^C	γ: 21.65	0.410	11.51	16.5
	α: 20.17	0.440	17.19	30.0
	α: 23.91	0.372	10.21	53.5

FT-IR data supports the XRD data where, in Figure 4D, the FT-IR data shows how the chemical functional group changes occurred as nanofibers were thermally treated. For example, the amide VI (*α*-phase) peak at 686 cm^-1^ is present in the pristine sample and as the heat treatment temperatures increased, this peak became sharper and more well-defined. From this peak shape change, we can conclude that a significant phase change occurred from *γ* to *α*. Thus, by comparing the structures of pristine nanofibers to heat-treated samples, it was determined that polymer annealing results in an increase of *α*-phase and the piezoelectric effect is lowered.

### 3.4 Wash durability testing for smart wear application

To evaluate the applicability of Nylon-6 nanofibers to be used for smart clothing, the mechanical durability of the material needed to be established. In Supplementary Figure S9, the V_33_ value as a function of strain was assessed by cantilever device testing. Initially, a strain of 0.017% generated 0.35 V which improved with an increase in strain. A maximum V_33_ of 1.74 V was observed for strain values of 0.033% or more. Therefore, it can be predicted that the V_33_ change will increase when the elongation change is large, and this result is expected to generate an effective piezoelectric effect from the movement of the human body for smart clothing applications. The stability of Nylon-6 nanofibers with F97 was confirmed as seen in Supplementary Figure S10 where the V_33_ change of Nylon-6 nanofiber was observed at 15-day intervals for 45 days. The same values of the initially measured V_33_ were continuously observed (Supplementary Figure S10A), and a stable waveform was observed without changing the V_33_ signal shape (Supplementary Figure S10B, C). This confirms that when Nylon-6 nanofibers are to be used as clothing, the piezoelectric properties would be stable.

**FIGURE 5:**
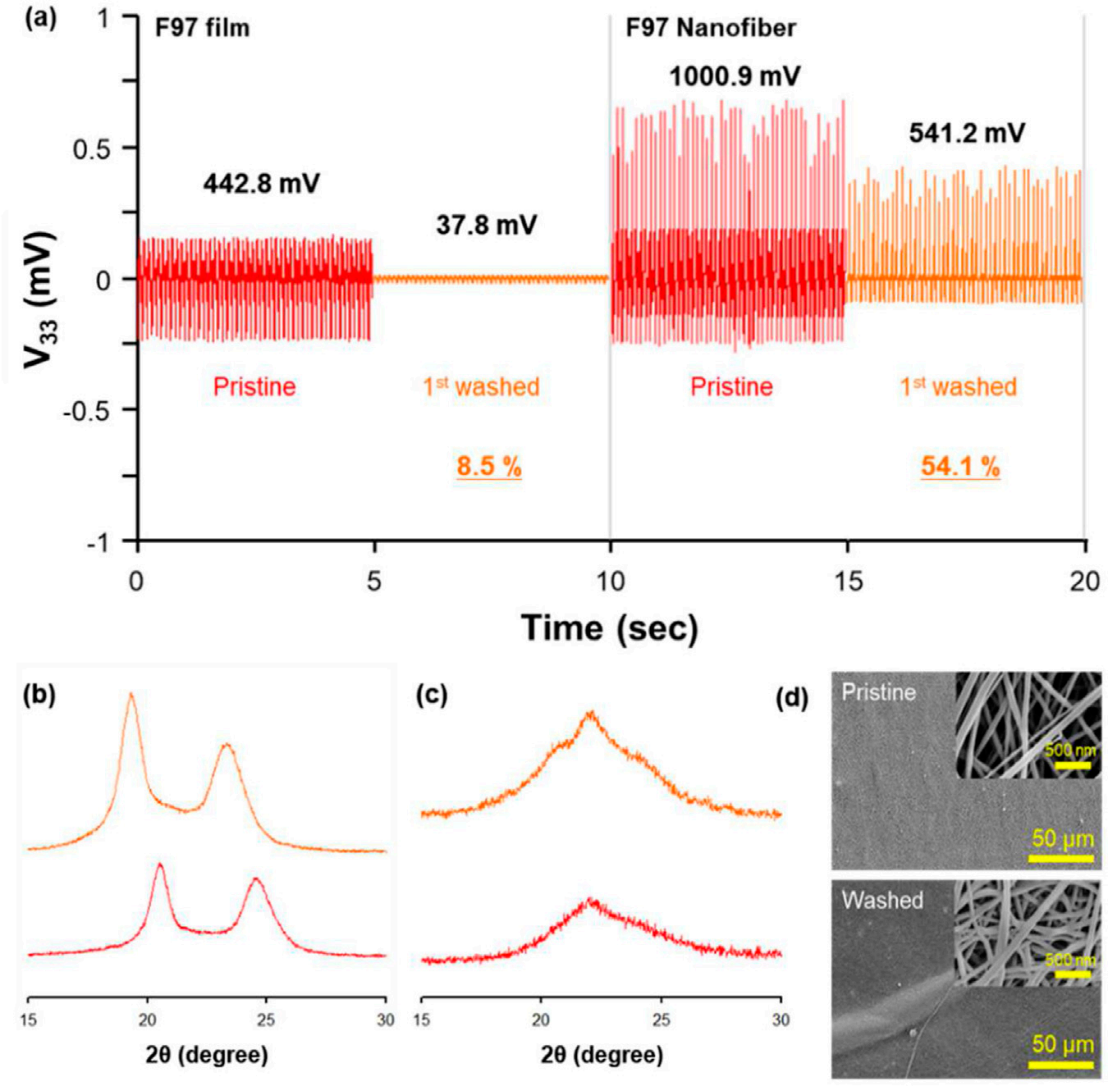
**(A)** Comparison of V_33_ change of Nylon-6 film and F97 by washing. XRD patter of **(B)** Nylon-6 film and **(C)** F97 according to wash. **(D)** SEM image of F97 before and after washing.

Lastly, to test the capability of piezoelectric properties after washing, the change in V_33_ value was investigated (Figure 5). The general Nylon-6 film had a V_33_ of 0.44 V before washing, but 91% of the V_33_ was removed after single washing. This is due to the crystal structure of the Nylon-6 film becoming significantly changed as confirmed by XRD in Figure 5B. In comparison, the V_33_ of the Nylon-6 nanofiber that initially generated 1 V was reduced by 45.9% to 0.54 V after first wash due to the change of crystalline phase and nanofiber swelling (Figures 5C, D). This indicates that nanofibers exhibit greater durability against crystalline changes during washing compared to conventional films. This is because the dense network of tens of thousands of fibers formed in the electro spinning process is less susceptible to damage from washing than a simple film. This effect is likely due to size impact. The detailed XRD analysis results are summarized in Table 5. Most interestingly, the Nylon-6 nanofibers did not lose their piezoelectric properties even after washing (Zeng et al., 2014; Nair et al., 2006). If stable nanofibers after washing are realized then additional research on Nylon-6, such as structural improvement and surface modification, could yield an even-numbered Nylon as an optimal material for supplying power to smart wear.

**TABLE 5:** Peak Location and *d*-Spacing Based on XRD of the N6 film and nanofibers depending on the wash.

Type	Peak location (2θ, °)	*d*-spacing (nm)	Grain size (nm)	Area integrated % (%)
F97 Film	γ: 21.70	0.409	3.64	31.2
	α: 20.51	0.433	20.03	30.9
	α: 24.62	0.361	12.17	37.9
Washed F97 Film	α: 19.33	0.459	16.82	38.1
	α: 20.43	0.434	4.55	24.4
	α: 23.39	0.380	10.73	37.5
F97 Nanofiber	γ: 21.04	0.403	6.55	61.7
	α: 20.66	0.430	5.49	10.8
	α: 23.84	0.373	5.56	27.5
Washed F97 Nanofiber	γ: 22.04	0.403	9.27	40.2
	α: 20.20	0.439	6.41	27.4
	α: 23.82	0.373	5.05	32.4

## 4 Conclusion

Electrospun Nylon-6 nanofibers were fabricated for a systematic study of size-dependent piezoelectric properties. It was determined that, for Nylon-6, crystallinity could be altered depending on the solvents used, with a 97% formic acid solution producing a piezoelectric, washable Nylon-6 product. While the concentration of the polymer had the greatest effect on nanofiber production, it was shown that the production of beads and clumps decreased as the purity of the solvent increased. Through DOE analysis, optimal conditions for Nylon-6 nanofiber production have been obtained, generating an average of 1.6 V. The ability to optimize piezoelectric voltage output through precise fine-tuning of the electrospinning process for Nylon nanofibers, combined with the impressive retention of these properties even after washing, highlights significant potential for future piezoelectric applications. This advancement positions Nylon nanofibers as an economical and durable choice for next-generation energy-harvesting materials, such as smart wearable clothing.

## Data Availability

The original contributions presented in the study are included in the article/Supplementary Material, further inquiries can be directed to the corresponding author.
