# Supplementary material for: Low-cost high performance piezoelectric fabrics based on Nylon-6 nanofibers
Source: Front Chem. 2024 Dec 4;12:1525034. doi: 10.3389/fchem.2024.1525034 (PMC11652212; doi:10.3389/fchem.2024.1525034)
Supplement: Supplementary file 1 [file DataSheet1.docx]

Supplementary Material


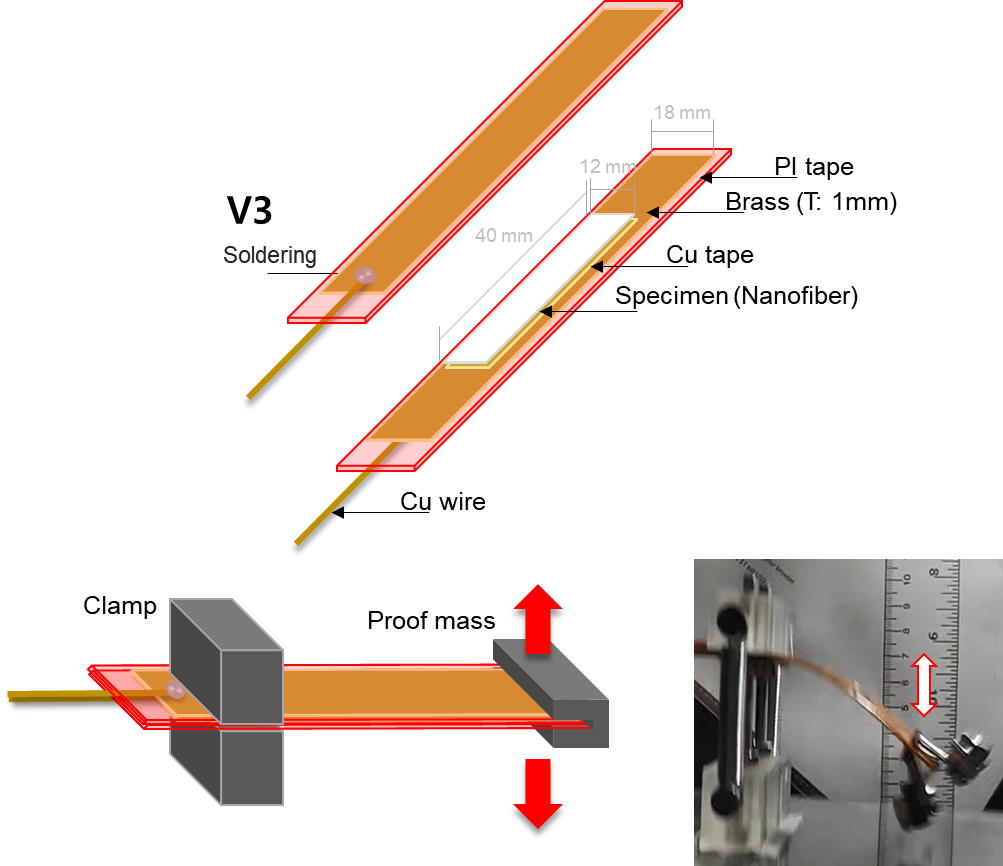


**Supplementary Figure 1.** Schematic of cantilever test for get a V_33_ value.


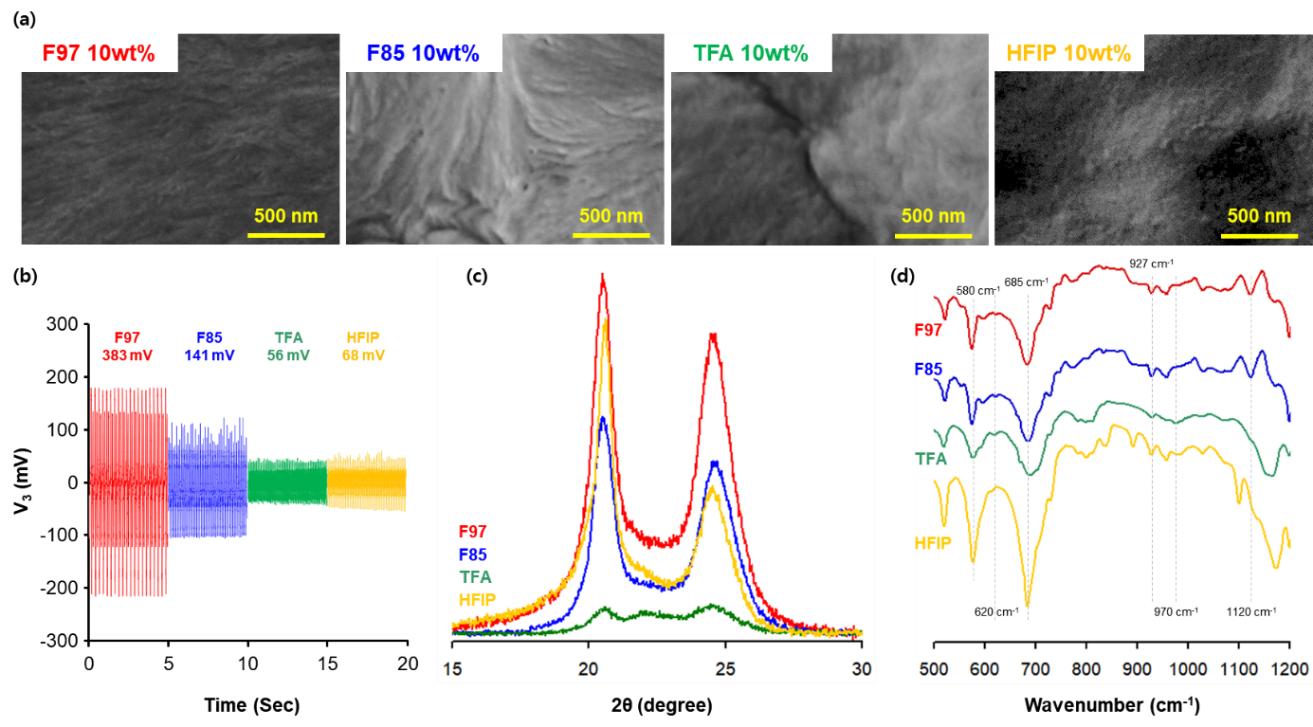


**Supplementary Figure 2.** (a) SEM of Nylon-6 film surfaces with different dissolved solvents; (b) V_33_ of Nylon-6 film with various solvents; (c) XRD patterns and (d) FT-IR spectra of Nylon-6 film.


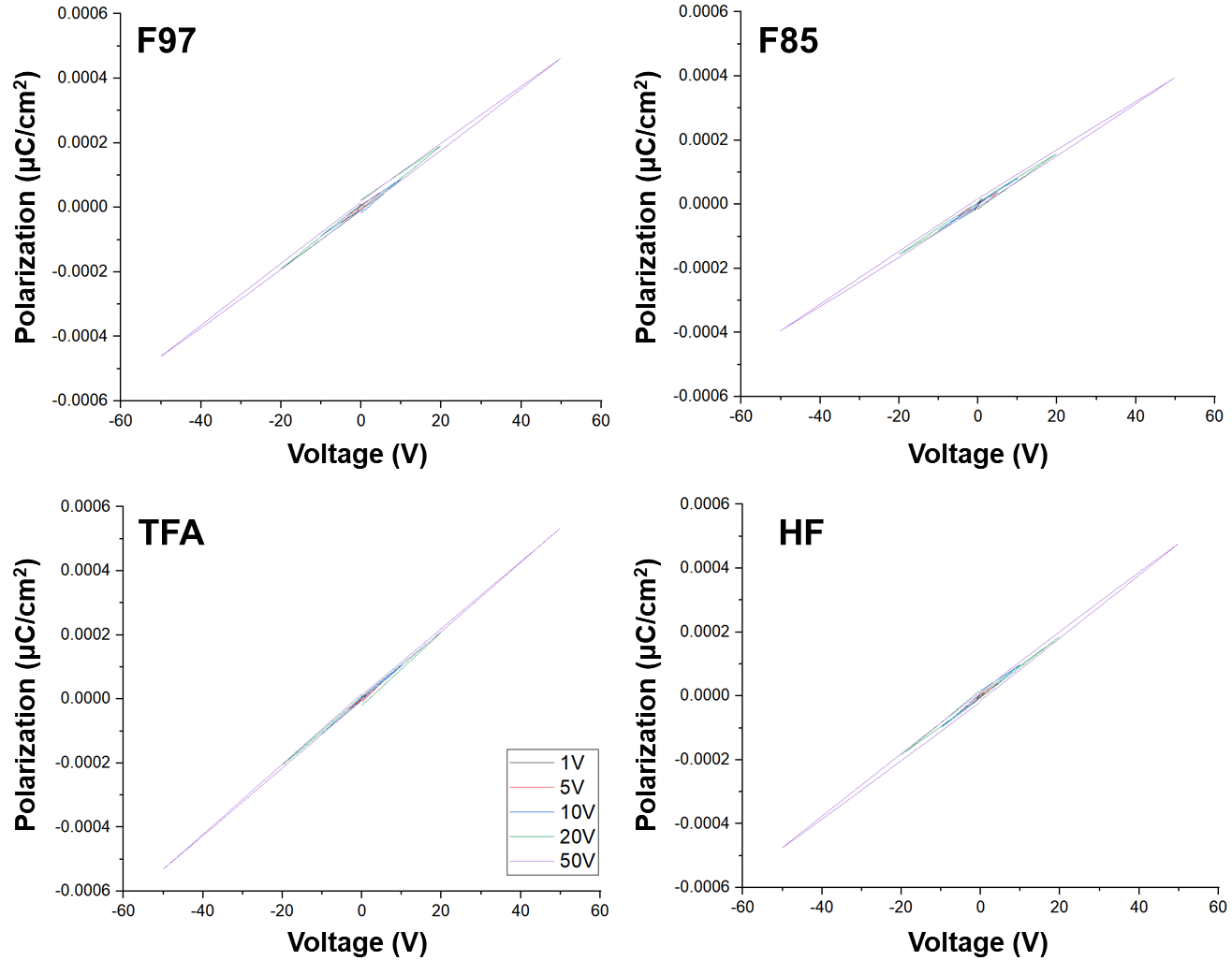


**Supplementary Figure 3.** (a) SEM of Nylon-6 film surfaces with different dissolved solvents; (b) V_33_ of Nylon-6 film with various solvents; (c) XRD patterns and (d) FT-IR spectra of Nylon-6 film.


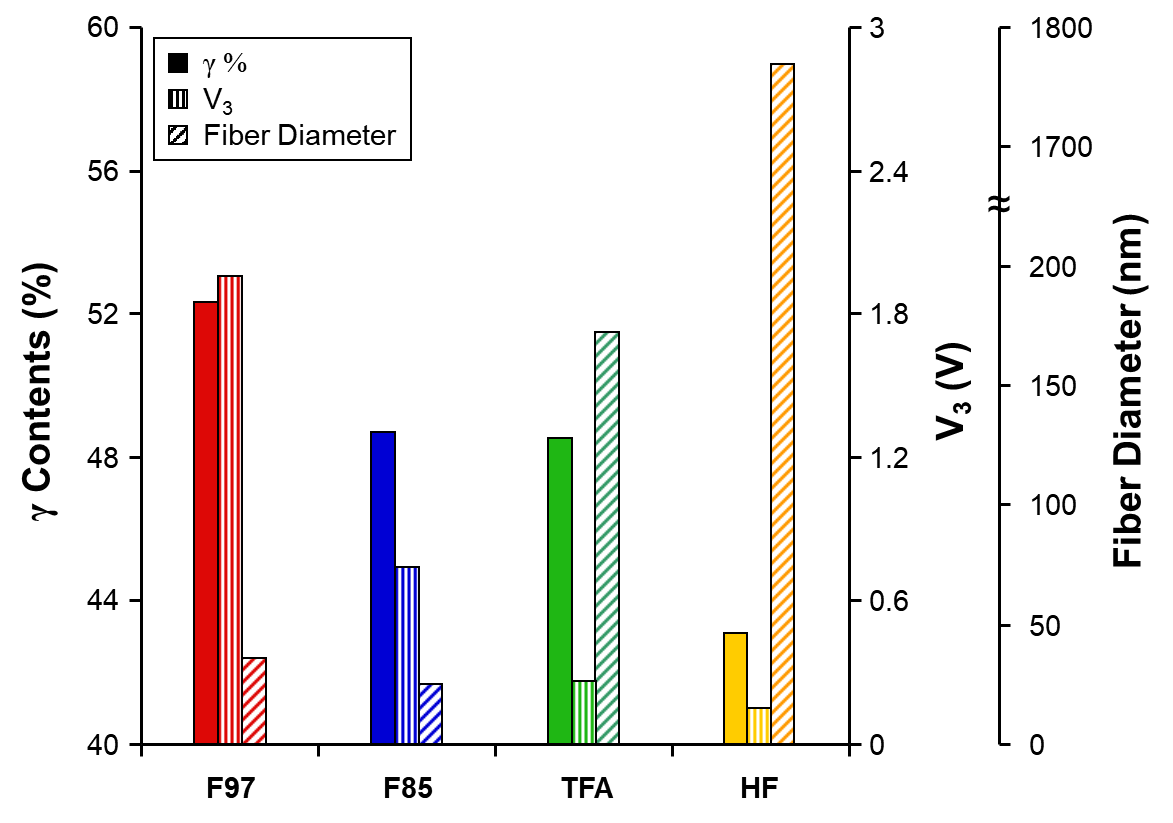


**Supplementary Figure 4.** Relationship of gamma contents, V_33_ and fiber diameter s of Nylon-6 nanofiber with the different solvents.


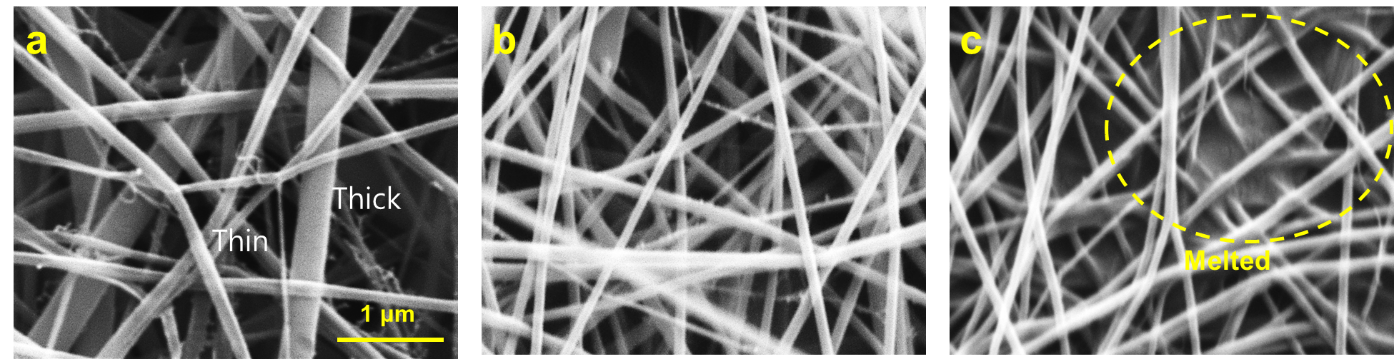


**Supplementary Figure 5.** Feed rate effect of Nylon nanofiber: Nylon-6/formic acid 97% (F97) 10wt% 23 voltage, 26 ^o^C, 40 RH% (a) feed rate: 0.1 ml/h; (b) 0.4 ml/h; and (c) 1 ml/h.


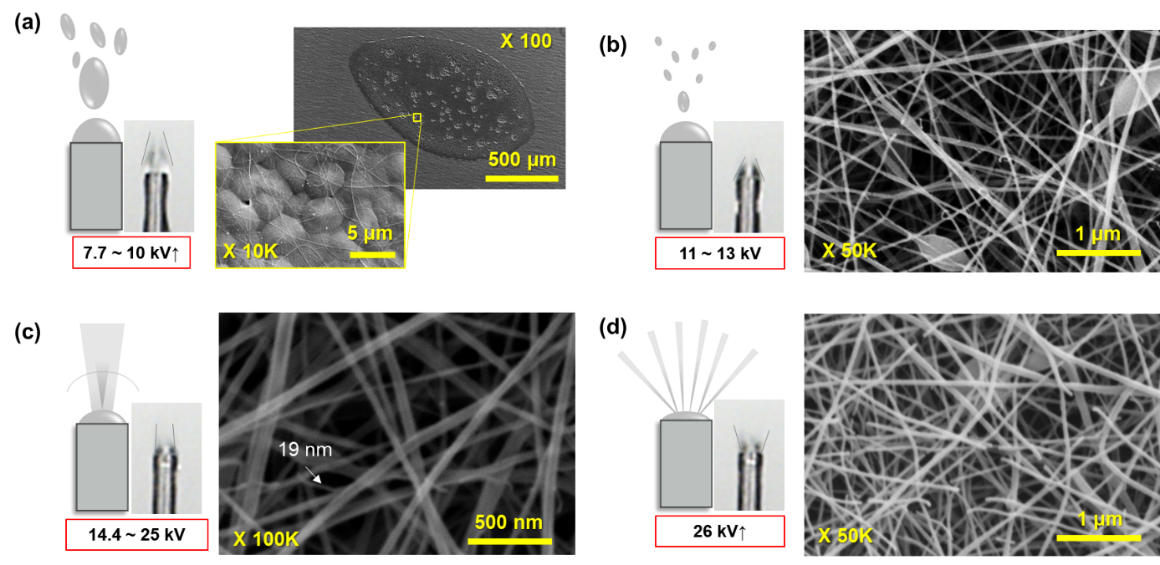


**Supplementary Figure 6.** Voltage effect of F97 nanofiber: 10 wt% 0.3 ml/h, 26 ^o^C, 40 RH%, (a) 7.7 ~ 10 kV; (b) 11 ~ 13 kV; (c) 14.4 ~ 25 kV; and (d) 26 kV or more.


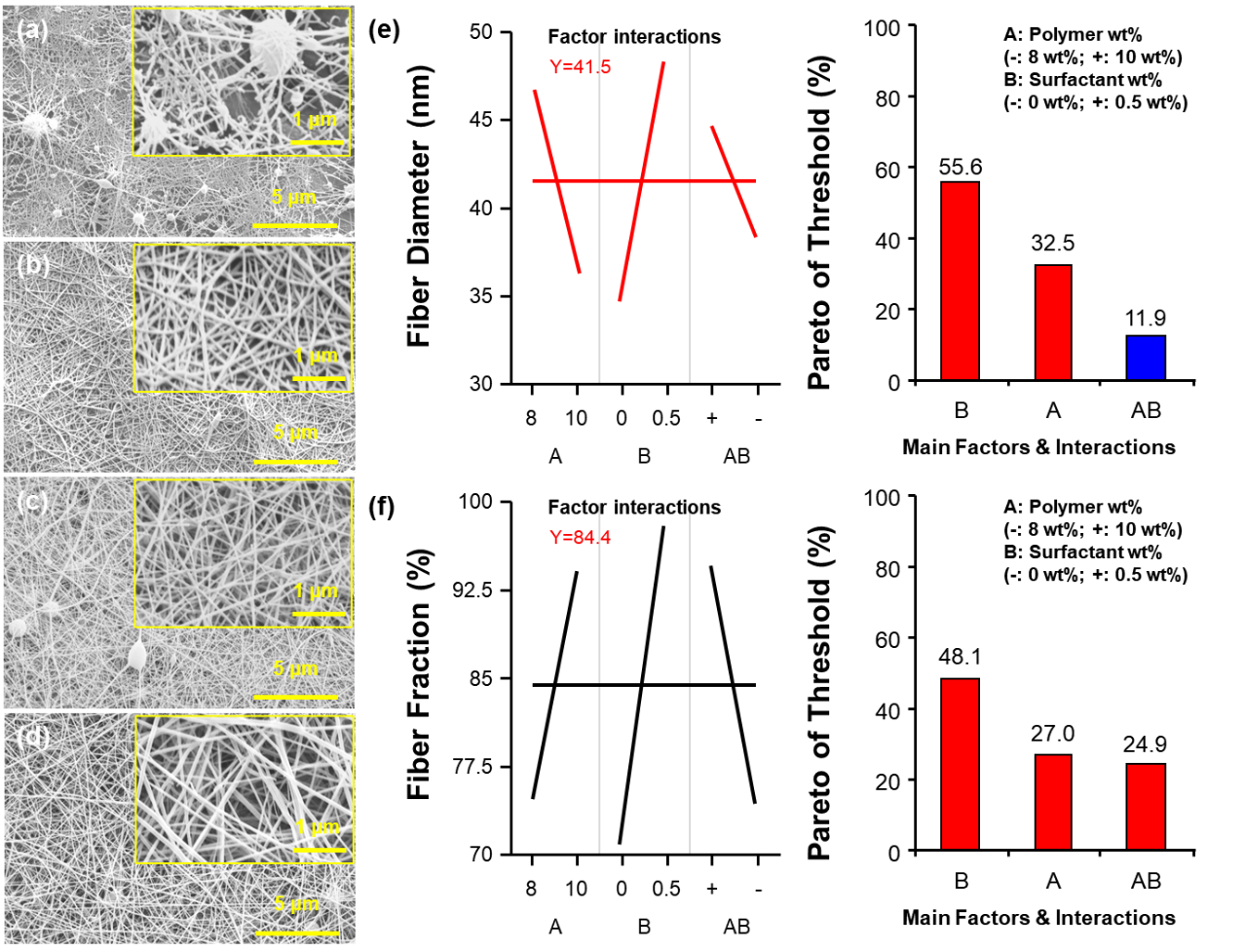


**Supplementary Figure 7.** Effects of electrospinning solution properties on the morphology of Nylon-6 nanofibers. Respectively, of the electrospinning solution properties (see Supplemental table 4) SEM images of (a) 1, (b) 2, (c) 3, and (d) 4 case for Nylon-6 nanofibers electrospun. Design-of-experiment (DOE) factor analysis and Pareto of threshold on (e) fiber diameter and (f) fiber fraction.

**Supplementary Figure 8.** Real V_33_ result of F97 nanofiber depending on the fiber fraction and average diameter of fiber


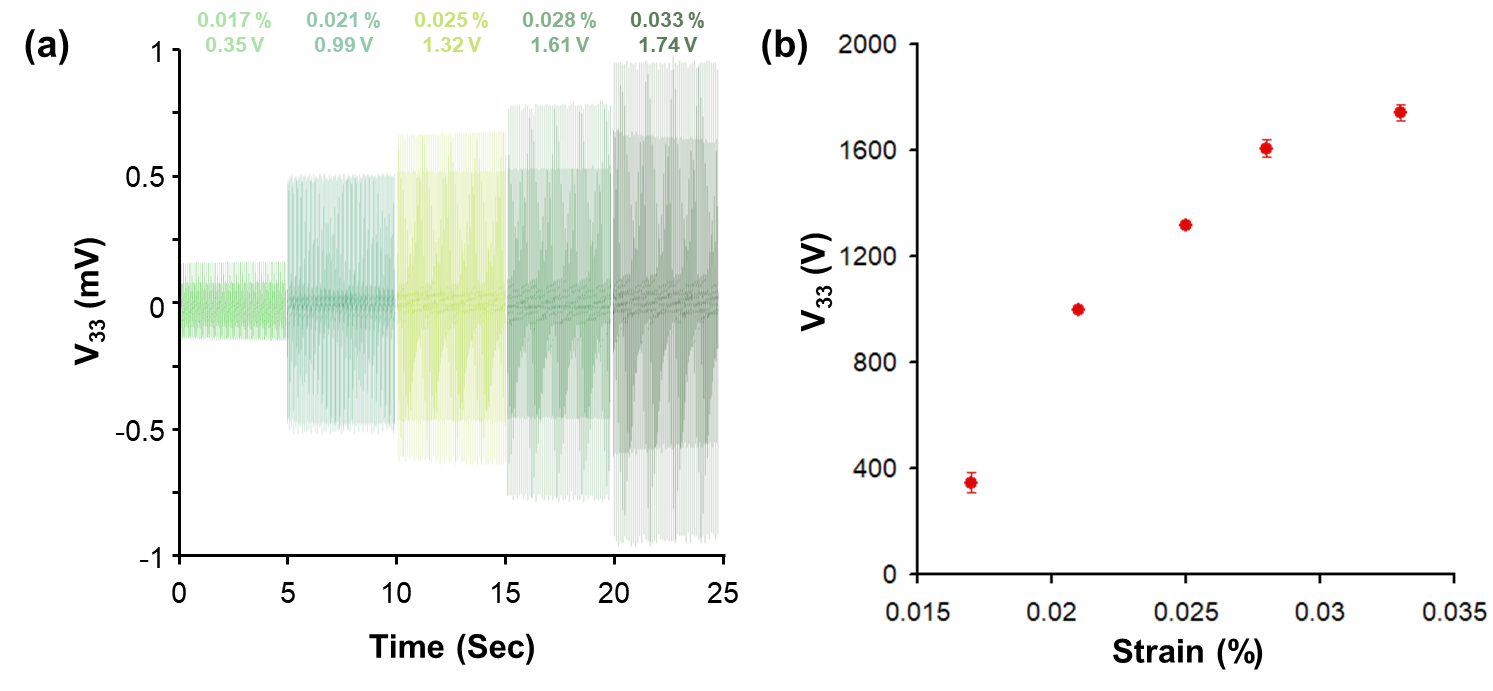


**Supplementary Figure 9.** (a) V_33_ of Nylon-6 nanofiber with various strain. (b) Average V_33_ as a function of average nanofiber strain.
